# Microbial biofilms and the human intestinal microbiome

**DOI:** 10.1038/npjbiofilms.2015.5

**Published:** 2015-03-25

**Authors:** Willem M de Vos

**Affiliations:** 1 Research Programme Unit Immunobiology, Department of Bacteriology and Immunology, Faculty of Medicine, University of Helsinki, Helsinki, Finland; 2 Department of Veterinary Biosciences, University of Helsinki, Helsinki, Finland; 3 Laboratory of Microbiology, Wageningen University, Wageningen, The Netherlands

## Abstract

Since early life we are colonised by a myriad of microbes that make up our microbiome. This colonisation process starts at birth or even before, when the virtually sterile baby encounters new microbial environments. It is likely that at this time or at later moments in life, microbial communities are met that have high-level structures with a temporal and spatial organisation, termed biofilms. This perspective will focus on these biofilms and the microbes in the intestinal tract as these are the most numerous in the human body, are found in luminal and mucosal locations, and have a great impact on human health and disease.

## Introduction

Recent metagenomic and other high-throughput studies have provided new insight in the structure, function and dynamics of the human microbiome, the collective term for human microbes and their genomes.^1,2^ These have provided molecular support for the notion that the human body is home to a myriad of microbes that colonise its cavities in time and space. Several of those contain complex microbial communities that are not planktonic but have higher order structures termed biofilms, microbial populations embedded in complex, self-produced polymeric matrices, adherent to each other, and surfaces or interfaces.^3–5^ An important reason to study these multispecies biofilms is that sessile cells often have a radical different physiology than planktonic cells, among others resulting in enhanced antimicrobial resistance, virulence and other functions, often related to quorum sensing.^5^

The first multispecies biofilms were observed more than 300 years ago by Antonie van Leeuwenhoek, when studying his own teeth and those of another person with limited oral hygiene.^6^ In more recent studies, the oral microbiome was found to be predominantly located in biofilms and has been characterised extensively for its involvement in dental caries, periodontitis and oral cancer.^7^ Considerable attention has also been given to biofilms that are found in a medical setting and colonise therapeutic devices, such as implants, tubes or stents, often causing various types of chronic infections with considerable clinical impact.^4^ These and other diseases, such as cystic fibrosis, endocarditis or prostatitis, are associated with microbial biofilms, which are often multispecies consortia with complex networks, special evolutionary forces and frequent horizontal gene transfer, and hence are increasingly difficult to treat.^5^

The intestinal tract contains the most densely colonised ecosystem of the human body, and it is this microbiome that has developed into a paradigm of beneficial interactions with its host.^8^ The major functions of the intestinal microbes have an impact on its holder in a variety of ways. The metabolic functions have a direct impact and include the conversion of non-digestible food components such as complex sugar polymers into short-chain fatty acids, the degradation of toxic compounds and the production of vitamins. However, the signalling functions of the intestinal microbiome and its products are increasingly recognised for their importance and modulate the host’s immune system or influence host development and physiology. This explains why deviations in the intestinal microbiome have been associated with dozens of diseases, varying from inflammatory bowel disease to type 2 diabetes and colorectal cancer.^9^ However, the complexity of the intestinal microbiome is unprecedented. Although over 1000 microbial, mostly bacterial and anaerobic, species have now been cultured from the human intestine, the majority of its microbial diversity has yet to be grown in pure culture.^10^ In addition, the intestinal microbiome is highly personalised and contains over 10 million genes.^11,12^ For simplicity and convenience, most studies have focused on the faecal microbiome, not taking into account the spatial organisation of the intestinal communities, such as those associated with lumen, food particles or mucosa. Hence, intestinal biofilms have received only limited attention and if so mainly associated with disease.^13^ This is in contrast with some animal studies where biofilms are essential, such as in rodents that have a fore-stomach biofilm consisting of host-specific lactobacilli.^14^ However, biofilms or biofilm-like structures in the human intestine may have a great impact on the function of the intestinal microbiome and its interactions with the host as will be summarised here.

## Early life colonisation and biofilms

The main microbial colonisation of the intestinal tract starts at birth when the virtually sterile baby encounters its environment and the first microbial biofilms. However, even *in utero*, microbial programming may occur as the placenta and amnion fluids of the mother may contain microbes and even intra-amniotic biofilms have been described.^15,16^ However, in healthy pregnancies there are various defense systems that prevent such colonisation, such as the production of the SALSA agglutinin that is highly abundant in amnion fluid and dominate the meconium.^17^ On delivery, the virtually sterile baby encounters the first microbial biofilms, possibly already in the delivery canal where biofilms have been reported in bacterial vaginosis, although this presents an increased risk of early pregnancy loss.^18^ Subsequently, the oral cavity of the happy parents, family members or other humans, or even pets, may be a source of biofilm microbes that come into contact with the newborn. More unique for the mother are the microbial communities of the breast that are transferred to the baby via milk as a delivery and nutritious selection system.^15^ However, not so well characterised is the faecal-oral transfer either directly or via other body parts or surfaces. This is quantitatively the most important colonisation route, simply as the faecal microbes may reach numbers of over 10^11^ cells per gram and hence are found everywhere in spite of our present hygienic procedures. The observation that about half of the faecal microbes are dead or damaged does not preclude this route since it is all a number game.^19^ As the majority of the intestinal microbes are anaerobes, there should be simple and evolutionary preserved systems to transfer the faecal microbiome to the newborn. Here biofilms are likely to have a role, as it has been shown that their structure and composition protect cells from desiccation and other environmental stressors.^20^

## Biofilms in the colonic microbiome

There have been various reviews supporting the presence of biofilms in the colonic microbiome but only few original studies have been published and these date from a decade ago or more.^21–23^ Most of the experimental approaches relied on microscopic observations of food particles covered with microbes and *in vitro* fermentation studies or continuous flow systems with plant fibre polymers. These raised some controversy, most likely because the used approaches did not have the appropriate sensitivity. In one study it was reported that bacterial populations that were strongly adhering to particulate matter in stools were phenotypically similar in composition to unattached communities.^24^ However, in later studies it was found that the species composition of the particle-associated and liquid phase communities was clearly different.^25,26^ A specialised set of microbes was found to be associated with the solid food particles of wheat bran and resistant starch, and these mainly included Ruminococci, notably *R. bromii*-like bacteria that accounted for over 10% of the solid microbiome. This is of interest, as in degradation studies using ^13^C-labelled starch and RNA-SIP, a trophic chain was identified consisting of *R. bromii*, digesting starch into acetate, and *Eubacterium rectale,* converting the produced acetate into butyrate.^27^ Obviously, trophic chains are needed to degrade the complex plant polymers in the colon, and it is known that multispecies biofilms with specific spatial organisation are essential for syntrophic conversions in another anaerobic environment.^28^ Hence, it is time to address the structural composition of the intestinal microbiome in time and space to advance our insight into its functions beyond a mere compositional or metagenomic analysis.

## Mucosal biofilms—do they exist?

The communication between intestinal microbes and host is increasingly perceived as of eminent importance and is mediated by microbial compounds, such as metabolites, cell components and other small molecules that signal to host receptors, from which the TLRs or GPRs are best studied.^8^ However, direct interactions with the mucosal surfaces are crucial as they allow specific intestinal microbes to be close to the intestinal cells, which is relevant as diffusion distance and barriers have a role. Hence, it has been known for a long time that colonic mucosal biopsies contain a different microbial composition than the luminal or faecal samples.^29^

Various microbes have developed systems to bind to mucin, the glycoprotein that is the major component of mucosa. These do not only include pathogens that couple this binding to invasion strategies but also commensals, such as *Lactobacillus rhamnosus* and related bacteria that have mucus-binding pili allowing them to act on a distance.^30^ However, biofilms as reported for these and other *Lactobacillus* spp. in the non-secretory, stratified squamous epithelia, such as in the fore stomach of or the chicken crop, have not been observed in the human intestine.^31^ This may be due to fact that the mucus layer of the human intestinal tract is very dense. As most microbes are found in the lower intestinal tract, several studies have focused on the colon and it appeared that the human colonic mucosa is ~500 μm in size and thicker and faster growing than that of the mouse colon. Moreover, the human mucosa grows with an astonishing speed of on average four microns per minute.^32^ This means that the mucosal surface is a challenging environment for an average microbe with a size of only a few microns, let alone for a biofilm that has to grow with that speed. This was already noted in the first comprehensive review of biofilms where the viscosity, movement and autochthonous microbial population were noted as factors that make colonisation and biofilm development very difficult.^3^ However, it cannot be excluded that there are sites in the colon where the mucosa is not as dense or fast growing, and where biofilms may be formed. This also holds for the upper intestinal tract that has much less studied and has a different microbiome with specific trophic chains.^33^ Whatever the architecture of the mucus layer is, the high colonic production rate rationalises the existence of efficient mucus degraders such as the specialist *Akkermansis muciniphila* that has developed specific interactions with the host and protects mice from diet-induced obesity.^34,35^

The high mucus production rate also complicates the study of the mucosal microbiome and its possible biofilms. Hence, early observations with mucosal layers of sudden death patients, where 60-μm- long microbes have been found at the top of the mucin layer, may be compromised due to the time needed for preparing the samples.^36^ However, the high mucus production capacity explains that the inner mucus layer of the human colon is largely sterile and only can be penetrated in diseases, such as ulcerative colitis.^37^ This is of interest, as in ulcerative colitis and other inflammatory bowel diseases, biofilms have been described covering the entire mucosa and entering the crypts, consisting mainly of *Bacteroides fragilis.* ^13^ In healthy subjects these biofilm-like structures were not found, or if detected, with a 100-fold lower amounts of cells. Hence, whether mucosal biofilms in healthy subjects really exist, or that the mucosal layer is completely sterile, is an unresolved question but needs to be addressed in view of the importance of the cellular signalling of the intestinal microbiome.

## Concluding remarks

Time has come to revisit the biofilms in the human microbiome. Microbial biofilms in disease have been well documented and important to study, also to challenge Koch’s paradigm as has been done previously.^38^ In healthy subjects, biofilms may be involved in delivering colonising anaerobes to the newborn baby, have an important role in the syntrophic degradation of polymeric substrates and may be involved in signalling to the host. A variety of novel approaches have been developed, such as dual transcriptomics and metaproteomics, to uncover the function mucosa and host,^39,40^ advanced microscopy, mass spectroscopy and the use of labelled substrates^27,41^ and a plethora of improved culturing systems.^10^ These can be applied to human microbiome to further develop mechanistic insight beyond the present associations of intestinal microbes with health and disease.
